# Supplementary material for: Exploration of designing an automatic classifier for questions containing code snippets—A case study of Oracle SQL certification exam questions
Source: PLoS One. 2025 Jan 9;20(1):e0309050. doi: 10.1371/journal.pone.0309050 (PMC11717213; doi:10.1371/journal.pone.0309050)
Supplement: S1 Appendix — (DOCX) [file pone.0309050.s001.docx]

# Appendix Nomenclatures to express the statistical results

Subscriptions:

- FRS: the factor of feature representation scheme.
- MLM: the factor of machine learning model.
- (x, y): the tuple expressing a level combination of the previous two factors. x is the level of the FRS factor, and y is the level of the MLM factor.
- T: TF-IDF scheme.
- W: Word2Vec scheme.
- F: FastText scheme.
- L: Logistics Regression model.
- M: Multinominal Naive Bayes model.
- S: Linear Support Vector Machine model.
- LS: Support Vector Machine model with the Radial Basis Function as the kernel.

Statistics:

- $m_{x}$: the mean of x level in a factor.
- $t_{x-y}$: t value for the contrast between x and y levels in a factor. For example, $t_{T-W}$ denotes the t value for the contrast between the TF-IDF and Word2Vec schemes; $t_{\left( T,S \right)-\left( F,M \right)}$ denotes the t value for the contrast between the (TF-IDF, SVM) and (FastText, MNB) groups.
- $F$: F value.
- $p$: p value.
- DF: degrees of freedom.
- $\eta^{2}$: the effect size of a factor in ANOVA.
- $d$: the effect size of t-test measured by Cohen's d.
