# Supplementary material for: Exploration of designing an automatic classifier for questions containing code snippets—A case study of Oracle SQL certification exam questions
Source: PLoS One. 2025 Jan 9;20(1):e0309050. doi: 10.1371/journal.pone.0309050 (PMC11717213; doi:10.1371/journal.pone.0309050)
Supplement: Attachment — Submitted filename: 3-Response to Reviewers.docx [file pone.0309050.s002.docx]

**A point-by-point response to the reviewers' comments**

The authors sincerely appreciate the reviewers for their valuable comments and suggestions for improving the manuscript quality. The following is a point-by-point response to the reviewers' comments.

# Comment 1

Line 47. “Bloom Taxonomy” A definition and/or explanation can help the reader.

## Response

1. Add a brief explanation of Bloom's Taxonomy.

2. Revise Bloom Taxonomy to Bloom’s Taxonomy in the manuscript.

## Revision Actions

## Before

For example, the questions could be classified using Bloom Taxonomy to assess the learner's cognitive ability, or they could be classified according to the exam topics to assess the learner's proficiency in specific subjects.

### After

For example, Bloom's Taxonomy is a classification of educational objectives to assess students' learning outcomes, including levels of remembering, understanding, applying, analyzing, evaluating, and creating. Questions could be classified using Bloom's Taxonomy to assess the learner's cognitive ability. Alternatively, questions could be classified according to the exam topics to assess the learner's proficiency in specific subjects.

Please see the marked-up annotated with “Comment 1”.

# Comment 2

- L88. The Literature Review section is very poor and poorly edited, unless considering the following sections, it seems more like a piece of the Introduction. Perhaps it would be better to integrate the following sections (L97, L113, and L131) into this one.

- TF-IDF/TFPOS-IDF and subsequent. Acronyms must be introduced before using them.

## Response

- Follow the PLOS ONE’ style guide to set the level 1 and level 2 headings to avoid the misunderstanding of readers.
- Add an orientation paragraph to introduce the structure of the literature review section.

## Revision Actions

The added orientation paragraph before the start of the first subsection in the literature review:

“Since the datasets, feature representation schemes, and machine learning methods impact the question classifiers, the remaining is divided into three subsections: Dataset, Feature Representation and Word Weights, and Machine Learning Methods.”

Please see the marked-up annotated with “Comment 2”.

# Comment 3

- (L88, Literature Review) TF-IDF/TFPOS-IDF and subsequent. Acronyms must be introduced before using them.

## Response

- Revising according to the comment.

## Revision Actions

Please see the paragraphs annotated with “Comment 3”.

# Comment 4

- L97. “Training Data Set” Perhaps, is "Classification Schemes" a more appropriate title?

## Response

- We revise the subsection title to "Dataset" to reflect the content better. The Dataset subsection reviews the datasets used in the question classification literature to learn the knowledge domains and the classification frameworks used to develop the question classifiers in the literature.
- Understand the characteristics of the dataset is important because they impact the classifiers' performance.

## Revision Actions

- Modify the subsection title from “Training Data Set” to “Dataset”.
- Modify the affected contents in the Literature Review section.

# Comment 5

- L99. “a data set in a domain is challenging to apply to another domain” Is this transfer learning or fine-tuning?

## Response

- The classifier developed from a data set in a domain is challenging to apply to another domain.
- The main reason is that question banks in different fields have different characteristics.
  - The question bank of language courses mainly consists of textual descriptions. The question bank content of mathematics courses mainly focuses on formulas.
  - The question bank of programming language courses is mainly based on instructions.
  - Moreover, even within the same discipline, question banks have significant differences due to different chapters.
- At this point, whether using transfer learning or fine-tuning for model training between different question banks is a great challenge.

## Revision Actions

No revision action is taken for the comment.

# Comment 6

- Table 1. The alignment of the lines is unclear and makes reading complicated.

## Response

Revise the table to improve the alignment of the lines for better readability.

## Revision Actions

Please see the revised table 1 annotated with “Comment 6.”

# Comment 7

- L118. TFPOS-IDF needs a short explanation. The same for ETF-IDF and ETFPOS-IDF.

## Response

Revising according to the comment.

## Revision Actions

Please see the revised paragraphs annotated with “Comment 7” in the literature review.

# Comment 8

- L127. Is E-TFIDF a typos or a new method?

## Response

The abbreviation of Enhanced TF-IDF has been changed to E-TFIDF.

## Revision Actions

Replace “ETF-IDF” with “E-TFIDF” in the manuscript.

# Comment 9

- L166. This section title does not seem necessary.

## Response

Revising according to the comment.

## Revision Actions

- Remove the subsection title “Question Classification Criteria and Assumptions”

- Revise a sentence to connect with the context. Please see the marked-up annotated with “Comment 9”.

# Comment 10

- L192. “manually labeled the questions” Is that the second column of Table 4?

## Response

- "manually labeled the questions" means that the questions were manually annotated with the topic identifications.
- Table 4 lists all the topic identification numbers, names, and the number of questions under each topic.
- The sentence is revised to clarify this meaning.

## Revision Actions

- The revision is annotated with “Comment 10”.

# Comment 11

- L221-223. These three lines should be better contextualised.

## Response

Extend the paragraph to elaborate on the process of using TF-IDF to vectorize a question.

## Revision Actions

Please see the marked-up annotated with “Comment 11”.

### Before

The words in the training dataset can be used to encode each question to generate a question vector. Let $V$ be the set of all words in the training dataset. The vector of question $j$ is $\vec{v_{j}}=\{f_{ij},i=1\ldots\left| V \right|\}$.

### After

The process of using TF-IDF to vectorize a question involves two steps. In the fitting step, the TF-IDF model is trained on the training dataset to build the vocabulary and calculate $idf_{i}$ for each word in the vocabulary. Then, in the transform step, the learned vocabulary and the $idf_{i}$ values are used to encode a new question to a vector. The word frequency in the new question is multiplied by each word's $idf_{i}$ value to generate the question vector. Specifically, let $V$ be the vocabulary. Then, the vector of the new question $j'$ is:

$$\vec{v_{j'}}=\left[ tf_{1j'},\ldots,tf_{ij'},\ldots,tf_{\left| V \right|j'} \right]\times\left[ idf_{1},\ldots,idf_{i},\ldots,idf_{|V|} \right]^{T},$$

(1)

where $tf_{ij'}$ is the frequency of word $i$ in new question $j'$ and $i\in V$.

# Comment 12

- L234. “We generate the question embeddings by compositing the word embeddings.” How? This part needs a better explanation.

## Response

Revising the paragraph to explain how the question embeddings are generated by compositing the word embeddings.

## Revision Actions

- The revised paragraph is annotated with “Comment 12”. We also provide the before and after revised paragraphs in the following.
- Adjust the equation numbering affected by this revision accordingly.

### Before

We generate the question embeddings by compositing the word embeddings. This study averaged the word embeddings to create the question embedding. Let $\vec{v_{ij}},i=1\ldots n_{j}$ denote the word embeddings in question $j$, where $n_{j}$ is the number words in question $j$. Then, the question embedding is $\vec{v_{j}}=\sum_{i=1}^{n_{j}} \vec{v_{ij}}\times\frac{1}{n_{j}}$.

### After

We generate a question embedding by averaging the word embeddings for the question. Let $\vec{v_{i}}=\left[ e_{1},\ldots,e_{k},\ldots,e_{m} \right]$ denote the embedding vector for word $i$, where $e_{k}$ is the *k*-th element in the embedding vector, and $m$ is the embedding dimension. Additionally, let $V_{j}$ be the set of words in question $j$. Then, the embedding vector for question $j$ is:

$$\vec{v_{j}}=\sum_{i\in V_{j}} \vec{v_{i}}\times\frac{1}{\left| V_{j} \right|}$$

(2)

# Comment 13

- L287. “as follows: The recall” A capital letter should not be used after a colon.

- L289. “instances from TP and FN instances” Acronyms must be introduced before using them.

- L292. In equation (1), what does c represent?

## Response

- Revise the sentence to follow the correct punctuation rules.
- Introduce the acronyms before using them.
- Describe the meaning of c in the equation.

## Revision Actions

Please see the marked-up annotated with “Comment 13”.

# Comment 14

- L361. “the following nomenclatures” Consider whether to move it to a separate section or to the appendix.

## Response

- Add an orientation sentence to indicate that the appendix provides the nomenclatures for epxressing the statisical result.
- Move the nomenclatures to the appendix.

## Revision Actions

- Please see the marked-up annotated with “Comment 14”

# Comment 15

- Section Experiment Results and Discussion. The figures are of very low quality and not readable. In addition, tables could be added to better summarise the results.

## Response

- Improve the quality of the figures. Use the Preflight Analysis and Conversion Engine (PACE) diagnostic tool to validate the figure quality.
- Add a table summarising the results in terms of the three performance metrics. Sentences to describe the table are added accordingly.

## Revision Actions

- Please see the marked-up annotated with “Comment 15)
- The added contents are also provided as follows.

### After

Table 6 summarizes the experiment results for the three performance metrics. The factors FRS, MLM, and their interaction significantly affect the weight macro-average AUC with large effects. The classifier using the TF-IDF scheme with the LOGREG model achieves the best performance, with a mean of 93.812%. Next, in the metric of the weighted macro-average precision, FRS and MLM factors significantly affect the performance with large effect sizes and their interaction with the intermediate effect. The classifier with the TF-IDF schema and the SVM model performs best, with a mean of 72.574% on the weighted macro-average precision. Lastly, the factors FRS, MLM, and their interaction significantly affect the weighted macro-average F1-score with large effect sizes. The classifier using the TF-IDF scheme and the LOGREG model achieves the best mean, averaging 81.574% on the weighted macro-average F1-score. The following subsections present the details.

Table 6. Summary of the experiment results for the three performance metrics: weighted macro-average AUC (wAUC), weighted macro-average precision (wP), and weighted macro-average F1-score (wF1).

|  | Metric: wAUC(%) | | | | |
| --- | --- | --- | --- | --- | --- |
| Effects | F-Value | Eta Square | Magitude of the Effec Size | Best Level | Mean of Metric |
| FRS | 3423.550 | 0.656 | Large | TF-IDF | 92.924 |
| MLM | 757.710 | 0.388 | Large | LOGREG | 88.900 |
| FRS:MLM | 343.700 | 0.365 | Large | (TF-IDF, LOGREG) | 93.812 |
|  | Metric: wP(%) | | | | |
| FRS | 774.470 | 0.302 | Large | TF-IDF | 72.378 |
| MLM | 110.700 | 0.156 | Large | LSVM | 70.632 |
| FRS:MLM | 191.160 | 0.138 | Intermediate | (TF-IDF, SVM) | 72.574 |
|  | Metric: wF1(%) | | | | |
| FRS | 2329.630 | 0.565 | Large | TF-IDF | 61.468 |
| MLM | 1320.740 | 0.525 | Large | LOGREG | 57.929 |
| FRS:MLM | 217.990 | 0.267 | Large | (TF-IDF, LOGREG) | 63.545 |

# Comment 16

- L536-540. “TF-IDF scheme outperformed those with the Word2Vec and FastText schemes”, “with the TF-IDF scheme outperformed those with the Word2Vec and FastText” Unhelpful repetitions. This section should be better maintained.

## Response

Revise L536-540 to avoid repetitions.

## Revision Actions

Please see the marked-up annotated with “Comment 16”.

# Comment 17

- From L545 to L553. Can these results also be valid with applications that contain code from other languages, such as C++ or Java?

## Response

Since the C++ and Java programming languages have different lexical units, syntax, and structure from SQL, the preprocessing and feature extraction stages might need adjustments. As mentioned in the fourth point of the research limitations in the conclusion section, further studies are needed to apply the proposed method to code from other languages.

## Revision Actions

No revision actions are taken.

# Comment 18

- L556. “classifying the instances in the TagMyNews dataset.” It seems quite obvious since it is a different context. Perhaps it should be better explained.

## Response

Revise sentences to Elaborate on the implications of “classifying the instances in the TagMyNews dataset”.

## Revision Actions

Please see the marked-up annotated with “Comment 18”.

# Comment 19

- Check references section. For instance, who is the author-editor of the first entry?

## Response

Check the references section and ensure that all entries are correctly formatted.

## Revision Actions

Reform the references.

# Others

The manuscript has been proofread thoroughly again for grammar, spelling, and readability.
